# How Well Do ‘Catch-Only’ Assessment Models Capture Catch Time Series Start Years and Default Life History Prior Values? A Preliminary Stock Assessment of the South Atlantic Ocean Blue Shark Using a Catch-Based Model

**DOI:** 10.3390/ani12111386

**Published:** 2022-05-27

**Authors:** Richard Kindong, Feng Wu, Siquan Tian, Ousmane Sarr

**Affiliations:** 1 College of Marine Sciences, Shanghai Ocean University, Shanghai 201306, China; kindong@shou.edu.cn (R.K.); ousmanesarr218@gmail.com (O.S.); 2 National Engineering Research Center for Oceanic Fisheries, Shanghai Ocean University, Shanghai 201306, China; 3 Key Laboratory of Sustainable Exploitation of Oceanic Fisheries Resources, Ministry of Education, Shanghai 201306, China; 4 Key Laboratory of Oceanic Fisheries Exploitation, Ministry of Agriculture, Shanghai 201306, China; 5 Scientific Observing and Experimental Station of Oceanic Fishery Resources, Ministry of Agriculture, Shanghai 201306, China

**Keywords:** overfishing, management, CMSY++, bycatch species, ICCAT, BSP

## Abstract

**Simple Summary:**

Blue shark species are at the top of the list of captured bycatch sharks in most tuna and tuna-like fisheries. As a consequence, their populations have been declining due to overfishing; thus, there is a need for the assessment of their stocks to better understand blue sharks’ stock status. Most bycatch species lack sufficient data for traditional stock assessment models to be implemented. Blue sharks in the South Atlantic have been assessed in the past using a state-space production model. Given the development of new assessment models and the use of up-to-date data, their stock status was evaluated using a new state-space production model (CMSY++). We used different catch time series, abundance indices and priors to measure the intrinsic growth rate r to evaluate their influence on the outputs of CMSY++. We identified from many scenarios that the blue shark stock in the South Atlantic may be witnessing overfishing and is being overfished.

**Abstract:**

CMSY++, an improved version of the CMSY approach developed from Catch-MSY which uses a Bayesian implementation of a modified Schaefer model and can predict stock status and exploitation, was used in the present study. Evaluating relative performance is vital in situations when dealing with fisheries with different catch time series start years and biological prior information. To identify the influences of data inputs on CMSY++ outputs, this paper evaluated the use of a nominal reported catch and a reconstructed catch dataset of the South Atlantic blue shark alongside different priors of the blue shark’s productivity/resilience (r) coupled with different indices of abundance. Results from the present study showed that different catch time series start years did not have a significant influence on the estimation of the biomass and fishing reference points reported by CMSY++. However, uninformative priors of r affected the output results of the model. The developed model runs with varying and joint abundance indices showed conflicting results, as classification rates in the final year changed with respect to the type of index used. However, the model runs indicated that South Atlantic blue shark stock could be overfished (B2020/Bmsy = 0.623 to 1.15) and that overfishing could be occurring (F2020/Fmsy = 0.818 to 1.78). This result is consistent with the results from a previous assessment using a state-space surplus production model applied for the same stock in 2015. Though some potential could be observed when using CMSY++, the results from this model ought to be taken with caution. Additionally, the continuous development of prior information useful for this model would help strengthen its performance.

## 1. Introduction

The blue shark *Prionace glauca* is a highly migratory pelagic shark species with a circumglobal distribution found throughout all oceans in tropical, subtropical, and temperate waters [1,2,3]. It is particularly vulnerable as bycatch in longline fisheries and has a near-threatened status according to the International Union for Conservation of Nature and Natural Resources (IUCN) [4,5]. Blue sharks are usually captured from the surface to a depth of at least 600 m [1,6]. The blue shark is a top shark bycatch species for many commercial fisheries, especially those targeting tunas and tuna-like species in the Atlantic, Pacific, and Indian oceans [7,8,9,10,11]. Because large numbers of blue sharks are caught by various fisheries, the species’ stock status has become an issue of great concern for regional fishery management organizations (RFMOs), such as the International Commission for the Conservation of Atlantic Tunas (ICCAT), the Western and Central Pacific Fisheries Commission, and the Indian Ocean Tuna Commission. However, the IUCN reports that the population status of this species has been declining globally [5].

The continuous removal of blue sharks as well as other bycatch species, such as rays, marine mammals, and other sharks, may seriously alter the marine ecosystem structure [12]. Due to this growing concern, tuna RFMOs have made it their prime objective to use various approaches to effectively manage global marine resources to ensure balance in the marine ecosystem. Blue shark population trends have been obtained for populations in the three main oceans. This information was obtained from different assessment results from the North Atlantic [7], South Atlantic [13], North Pacific [10], South Pacific [8], and Indian oceans [14]. These assessments reported that different stocks were sustainably harvested; however, further assessment works need to be performed with much-updated data given that catches of blue sharks continue to increase.

The last stock assessment for the South Atlantic blue shark population was carried out in 2015 [7,13], and no assessment with updated data has been completed since. It is essential to track this stock’s population trend given the constant increase in catches, and also to test the effectiveness of newly developed assessment models on stocks such as the blue shark. The author of [13] used a state-space Bayesian Surplus Production (BSP) model to assess the South Atlantic blue shark stock. Ref. [7] also used a BSP approach for blue shark stock assessment in the Atlantic to assess the status of the same stock, but conflicting results were observed from the two models. However, these reports paved way for the implementation of other assessment methods for testing and comparison between models.

The data available for blue sharks in this region can be classified as moderate. Newly developed data-poor/-moderate assessment models such as the CMSY++ can be applied to evaluate the stock status of this threatened bycatch species. CMSY++ is an advanced state-space Bayesian method for stock assessment that estimates fishery reference points (maximum sustainable yield (MSY), Fmsy, Bmsy) as well as status or relative stock size (B/Bmsy) and fishing pressure or exploitation (F/Fmsy) from catch and (optionally) abundance data, a prior for resilience or productivity r, and broad priors for the ratio of biomass to unfished biomass (B/k) at the beginning, an intermediate year, and the end of the time series [15]. CMSY++ can incorporate, in addition to the catch time series, a wide variety of additional data and supplementary information in a rigorous Bayesian context that tends to reduce the dependency on prior information as much as possible, while remaining robust and thus usable in data-limited/-moderate situations [15,16].

This updated version of CMSY has recently been used by [16,17] to estimate the biomass and exploitation levels of some of the world’s commercially exploited species. These reports indicate the effectiveness of CMSY++ to evaluate species’ stock status, especially when combined with informative priors and some indices of abundance data. Some of the species analyzed using CMSY++ were cod, sole, European anchovy, yellowfin tuna, sardinella (round and Madeiran), and many others, as indicated in these reports [15,16,17]. These reports highlight the effectiveness of this model in providing basic reference points, particularly for stocks with no data available. Furthermore, the flexibility in the implementation of CMSY++ makes it interesting, as its performance can be improved whenever more data are available to develop priors from external sources [16]. This method can, at the same time, be applied either in a data-poor (catch-only) or a data-moderate (catch and catch-per-unit-of-effort (CPUE)) situation, making it suitable to assess fishery stocks worldwide, which will contribute to a much-needed better understanding of the world’s fisheries [18]. However, given that most assessment models are easily influenced by various input data, particularly data-poor and data-moderate models, it is also necessary to evaluate the performance of the CMSY++ model. Hence, the significance of evaluating the performance of the CMSY++ model, a newly developed state-space Bayesian method, using fishery data with different catch time series start years, CPUE indices, and biological prior information.

The last stock assessment of the South Atlantic blue shark indicated that any future increase in fishing mortality could cause the stock to be overfished and/or experience overfishing due to some unsustainable harvests witnessed in past years [7,13]. The recent updates on the blue shark in the Atlantic by the IUCN indicate that populations continue to decline, probably due to an increase in fishing mortality [4]. Therefore, up-to-date assessment studies using the best possible up-to-date data are necessary to better understand their population trend. This study used a recently developed stock assessment approach, CMSY++, to evaluate the South Atlantic Ocean blue shark population using available catch and abundance indices data. Catch data available from the ICCAT Task I nominal catch database and reconstructed catch presented in the ICCAT 2015 blue shark data preparatory meeting were used to test the influence of different catch time series start years on the outputs of the stock assessment model. Additionally, priors of the intrinsic growth rate r presented in the FishBase database and that were used in the last blue shark stock assessment were evaluated to identify their effects on the outputs of the assessment model. Furthermore, the present study also evaluates the effect on the model’s output of different abundance indices tested as additional runs in the model.

## 2. Materials and Methods

### 2.1. Study Area and Data Source

**Figure 1:**
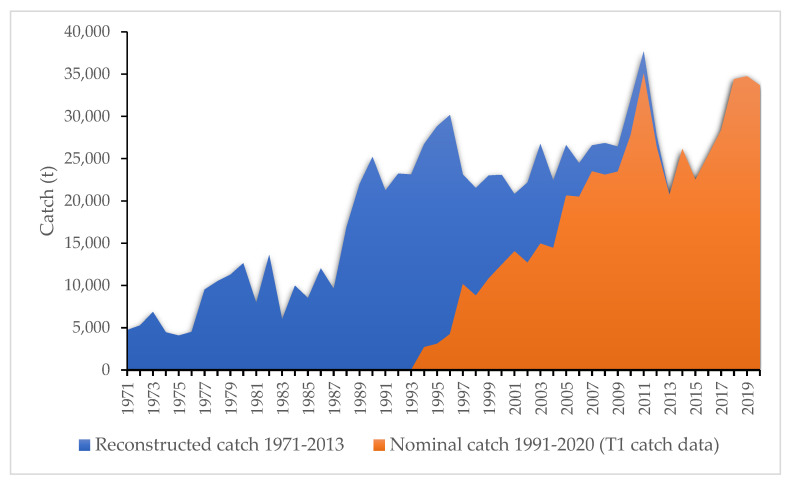
Historical reconstructed catches and nominal catch (T1NC) for the South Atlantic blue shark population. Catch reconstructed from 1971 to 2013; total used catch from 1971 to 2020. Nominal reported catch (T1NC) data from 1991 to 2020.

Blue sharks in the Atlantic Ocean are divided into two populations (north and south). This study focused on the South Atlantic blue shark population. Blue shark data used in this analysis were mainly catch data (Task I’s reported catch and reconstructed catches developed for blue sharks, ICCAT) and indices of abundance available for some countries. The catch data used in the present work were obtained from the 2015 blue shark data preparatory meeting report [19], which made estimations for many fleets and nations based on the best available information. This report presented a reconstructed catch dataset of the 2015 blue shark stock assessment [13,20]—different from the ICCAT Task I nominal catch data (T1NC) for the blue shark—using a ratio-based method. The general approach that was used to fill in some missing catches in the historical reconstructed catch dataset was the average catch between two adjacent years to capture the localized tendency. Figure 1 presents the catch time series for the reconstructed catch (catch series starts from 1971) used in the 2015 assessment and the nominal catch data (starting from 1991 (8 tons negligible)) for the South Atlantic Ocean blue shark. For this assessment study, we focused on both catch time series to evaluate the influence of this different catch time series information on the final assessment results. The blue shark data preparatory meeting also presented relative indices of abundance developed from the standardized catch-per-unit-of-effort (CPUE) time series available for some countries [19].

The abundance indices considered for this assessment were based on CPUE indices from longline fishery data for Uruguay (URG), Brazil (BRS), Japan (JPN), Spain (ESP), Taiwan, China/Chinese Taipei (CHTP), and combined CPUE (J_C). The same abundance indices were used in this study as those used in the 2015 blue shark assessment.

The biological information needed from this assessment was also obtained from the 2015 blue shark assessment report to ensure a sort of comparison of the present results to the previous ones. The bounds for the intrinsic growth rate (r) were obtained from FishBase [21] and the 2015 blue shark assessment report [13,20]. On one hand, the intrinsic growth rate bounds of (0.045–0.3) from r = 0.21 used in the 2015 blue shark assessment [20] were used, and on the other hand, the intrinsic growth rate r = 0.06 (0.045–0.10) was obtained from FishBase [21].

### 2.2. CMSY++ (CMSY and BSM)

The method CMSY++ is an innovative state-space Bayesian method that comprises two solid analytical methodological parts, both based on a modified Schaefer surplus production model: (1) the CMSY part, the method that treats catch-only data, and (2) the other part of the method, BSM (Bayesian Schaefer Model) that requires additional abundance data [15]. Furthermore, typical production models use catch time series data and indices of abundance data to estimate productivity. In its place, the CMSY++ method uses a catch dataset and a prior for resilience or productivity (r), abundance data (optionally), and broad priors for the ratio of biomass to unfished biomass (B/k) at the beginning, an intermediate year, and at the end of the time series. These inputs by CMSY++ are used to estimate biomass status or relative stock size (B/Bmsy), fishing pressure or exploitation rate (F/Fmsy), and related fishery biological reference points (MSY, Fmsy, Bmsy). The BSM applied in CMSY++, compared to other implementations of surplus production models, focuses on informative priors and also accepts short and fragmented years of abundance data [15]. In doing so, the CMSY++ provides an alternative assessment tool for situations where CPUE indices are not available or potentially unreliable.

The CMSY++ method is an improvement of the CMSY method presented in [22], which is an improvement of the Catch-MSY method of [23]. The main technical differences between CMSY++ and CMSY are the implementation of a fully Bayesian approach with MCMC (Markov chain Monte Carlo) modelling even when only catch data are available (CMSY analysis), and the prediction of default biomass priors from catch using an AI (Artificial Intelligence) neural network [15]. Additionally, in the previous version of CMSY, priors of r and k (carrying capacity) were uniformly distributed, whereas in the new CMSY++ an introduction of multivariate normal priors for r and k in log space is applied for both the CMSY and BSM methods. Hence, this allows the easy estimation of the ‘best’ r–k pair in CMSY and faster run times [15]. It is worth noting that CMSY++ addresses the overestimation of productivity at very low stock sizes (a general shortcoming of production models) by implementing a linear decline in surplus production when biomass falls below 1/4k.

### 2.3. Setting Ranges of Prior Parameters to Be Explored

Biological information is vital for properly informing the priors of CMSY++. The catch time series were derived from the 2015 blue shark data preparatory meeting report and updated blue shark’s T1NC data (up to 2020). Relative abundance indices for blue sharks consisted of standardized catch-per-unit efforts (CPUEs) for Japanese, Brazilian, Uruguayan, Spanish, Taiwanese, and Chinese longline fisheries.

The smallest and largest values of r obtained from FishBase ((0.045–0.1) [21] and the value of r used in the 2015 blue shark assessment (r = 0.21; ICCAT/SCRS/2015/014) were used to set the bounds of r (0.045–0.3) explored in CMSY++. In addition to this, CMSY++ was also run using the default approach, in which the resilience value available on FishBase was used to define the range of r. For the blue shark, resilience was estimated to be low using r = 0.21 [20] (Table 1), reflecting what we know of blue sharks: they are a slow-growing, late-maturing species that can produce many offspring (4–135 pups; FishBase) [21].

Regarding the range of depletion rates (B/k) at the start of the time series (1971), the stock is believed to be experiencing a low to medium depletion state, as can be seen in Figure 1 and the categories expressed in Table 1. Therefore, a wider initial depletion rate (B/k) of 0.4–0.8 was defined (Table 1 and specified in Table 2) based on ranges of depletion and resilience rates as stated in Froese et al. [15]. A larger range for the intermediate depletion rate was set to 0.2–0.9 for the year 1995 (reconstructed catch data) and 2011 for nominal catch data, to give the model more freedom. In order not to overly constrain the estimated stock trajectory, a wider range, between 0.1 and 0.7, was given as the depletion rate for the final year (2020).

Further model configuration and scenarios involved the choice of variances for the catch data (observation errors), CPUEs, and process errors. Process errors enable the population dynamics to deviate from the exact values given by the model, while still conforming to the assumptions of the model on average. The incorporation of process errors is useful for two reasons: (1) when the model is trying to fit an abundance index, process errors can reduce bias arising from lack of fit in a deterministic SRA whenever dynamics are poorly explained by catch history alone, and (2) with or without an abundance index (or other auxiliary information), the stochastic portion is necessary to obtain plausible uncertainty intervals in the final estimates [24,25].

### 2.4. Scenarios

A total of fourteen (14) scenario runs were performed; these scenarios were chosen from combinations of two input sources: catch time series (reconstructed versus T1NC reported catches) and different indices of abundance (Table 2). Seven runs comprised reconstructed catches from 1971 to 2020 and the other seven catches were from 1992 to 2020. Among the 14 runs, 1 run that included reconstructed catches (1971–2020), all input CPUE indices (standardized catch-per-unit effort (CPUE) for Japan, Brazil, Uruguay, Spain, and Taiwan, longline fisheries) and prior mean values was developed as a base case (BSH_ATS_JCPUE, Table 1: Run 14). Two scenarios were tested without any indices of abundance (only catch data, CMSY analysis) and twelve scenarios with catch and different indices of abundance (CMSY and BSM), with the aim to evaluate the sensitivity of the model to different assumptions regarding the changes in input data. The process error was set to 0.05 for all scenarios. The coefficients of variations (CVs) for catches and CPUEs were set to 0.2 and 0.15, respectively. The process error and CVs fixed in the present study were the same as those used in the Bayesian state-space surplus production model of the 2015 South Atlantic blue shark assessment [13,20]. Kobe plots were also presented. For the present study, we investigated the effect of time series start year, including catch time series starting in 1971 or 1992 (Table 2).

## 3. Results

### Effect of the Starting Year of the Catch Time Series and Choice of the Prior of r

The two options used to define the prior of r did change significantly, especially on runs with catch and CPUE data for both cases (Table 3 and Table 4). For the first case, when the prior of r obtained from FishBase was used, the r for all fourteen runs did not change significantly (Table 3) whereas, for the second case (r = 0.21), significant changes were observed between runs with only catch data and runs comprising catch and CPUE data (Table 4). The values obtained in the second case for all runs in Table 4 fell within the range presented in the 2015 blue shark stock assessment. For further analysis, the prior of r from FishBase was dropped given that this prior of r did not specify from which blue shark population (north or south) the value r = 0.06 was obtained. This study focused on the prior of r for the South Atlantic blue shark as used in the 2015 blue shark assessment (Table 4) so as to facilitate comparing the final results.

As seen in Table 3 and Table 4, setting the start year to 1971 or 1992 had no great influence on r when running CMSY++ with only catch data (CMSY) and no CPUE indices data (Runs 1 and 8). A slight increase in K was observed with the longer time series of catch data from 2027kt to 2065kt (Table 4). Similarly, we found that the start year of the catch time series had a negligible impact on the results for different runs when including CPUE in the catch data, except for the runs that included the Japanese CPUE (Runs 4 and 11: Table 4). The Japanese CPUE run with a short times series (Run 4: Table 4) had similar r values as runs with catch-only data. The runs with the Japanese CPUE examined across different start years had similar results, and presented extreme values for B/Bmsy (0.623, lowest value: Run 11, Table 4) and F/Fmsy (1.78, highest value: Run 11, Table 4). This difference observed in start years for Japanese CPUE and different runs with other CPUE indices may be attributed to differences in CPUE time series. The Japanese CPUE started in 1971, contrary to other CPUE indices, so might have influenced the results for different catch start years. The lack of influence of the start year observed for the other CPUE indices may be attributed to the catch rate before 1971 being lower than the current catch rate (Figure 1), and therefore having little impact on the estimates of K. Consequently, this low influence of start year observed for most runs advises the selection of the full dataset available; thereby, we adjusted our final model configuration to include the start year of 1971.

**Figure 2:**
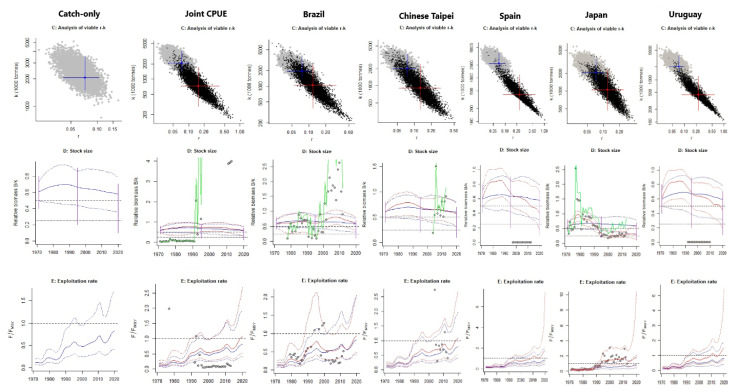
The output of the CMSY++ (CMSY and BSM models) using catch-only data (column 1) and the catch-per-unit effort (CPUE) indices (from columns 2 to 7) of the joint CPUEs of Brazil, Taiwan, China, Spain, Japan, and Uruguay. **Row 1** displays the r/k pairs found by CMSY catch-only and the BSM when using the full catch time series (from 1971 to 2020) with the dark grey points indicating possible r–k pairs found by the CMSY model and black dots indicating possible r–k pairs found by the BSM model. The blue crosses indicate the most probable r–k pairs found by the CMSY (catch-only) and the red crosses indicate the most probable r–k pairs found by the BSM and their 95% confidence limits. **Row 2** shows the estimated biomass relative to K (red), i.e., the CPUE data, scaled to the BSM estimate of Bmsy = 0.5 k, and the biomass trajectory estimated by CMSY in blue. Dotted blue and red lines indicate the 2.5th and 97.5th percentiles. The dots indicate the CPUE data scaled and corrected by BSM, and the green line indicates the uncorrected CPUE. Vertical purple lines indicate the prior biomass ranges. Horizontal dashed and dotted black lines indicate the 0.5 and 0.25 biomasses (BMSY), respectively. **Row 3** indicates the exploitation rate estimated from the BSM (red) and the CMSY (blue). The black horizontal–dotted line indicates where F/Fmsy = 1.

The Japanese, Spanish and Uruguayan BSMs indicated that biomass had dropped below Bmsy by 2020; meanwhile, CMSY (catch-only) for these three indices indicated biomass above BMSY. Spain’s BSM indicated biomass dropping below Bmsy by 2012, while this occurred from 2018 to 2020 for Uruguay, and from 1992 to 2020 for Japan, almost dropping to half of Bmsy, indicating a proxy of reduced recruitment (Figure 2, Row 2). Brazil, Taiwan, China and joint CPUE BSM estimates were in more accordance with the CMSY (catch-only) stock status (biomass) estimate than the other three indices and showed decreasing biomass, but were still higher than Bmsy by 2020 (Figure 2, Row 2). Further results indicated that the B/Bmsy values estimated by CMSY (catch-only) were more in line with the BSMs from Brazil, Taiwan and China, and joint CPUE with B/Bmsy > 1 indicated that the stock was not overfished (Runs 8, 10, 13–14: Table 3). Contrary to that, the Japanese, Spanish and Uruguayan BSM estimates indicated that the stock was overfished with B/Bmsy < 1.

**Figure 3:**
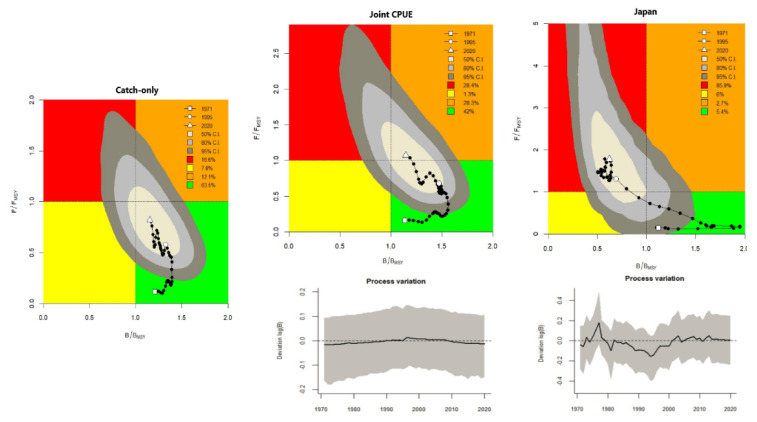
Kobe plots show the synchronized changes in exploitation (F/Fmsy) and the relative biomass (B/Bmsy). The orange area indicates healthy stock sizes that are about to be depleted by overfishing. The red area indicates that the stock is overfished and is undergoing overfishing. The yellow area indicates reduced fishing pressure on stocks recovering from still too low biomass levels. The green area indicates sustainable fishing pressure and a healthy stock size capable of producing high yields close to maximum sustainable yield (MSY). The lower panel figures represent process error deviations indicating changes in biomass diverging from the Schaefer model expectations (thick bold vs. dashed lines).

The Japanese BSM indicated that overfishing has been occurring throughout the last three decades. CMSY outputs indicated that exploitation rates begun relatively low, started rising in the mid-1980s, declined around 2010, and rose exponentially from 2016 to 2020, but remained below overfishing limits (Figure 1, Row 3). Apart from CMSY (catch-only) and the Brazilian BSM, the outputs from the other BSMs indicated that the stock was experiencing overfishing (F/Fmsy > 1) (Table 3; Figure 1, Row 3). Stock trajectories produced by each tested run were also evaluated. The CMSY (catch-only) run indicated a probability of 63.5% of the stock in the last year falling in the green area. When the abundance indices were put together (joint CPUE), BSM indicated that the probability of the stock falling in the orange area in the last year was 28.3%, and there was an 86% probability of this same stock falling in the red area in 2020 for the Japanese BSM (Figure 3). Furthermore, probabilities from the BSMs of Uruguay (61.5%) and Spain (72.6%) also indicated stock in the red area; the Chinese Taipei BSM indicated stock in the orange area (26.5% probability); and the Brazilian BSM showed that the stock in the last year fell in the green area (54.3%).

The process variation figures show the deviation between deterministic expectation (surplus production minus catch) and stochastic realization (after adding process errors); a strong deviation of the bold curve from the dashed line indicates that changes in biomass diverge from the Schaefer model expectations, perhaps due to the CPUE not properly describing the abundance or the priors being mis-specified (Froese et al., 2021). Slight deviations could be observed for the Japanese CPUE run and an insignificant change was observed when the CPUEs were combined.

## 4. Discussion

The present study shows that the start year catch time series had an insignificant influence on the final results, whereas the assignment of intrinsic growth (r) greatly affected the outputs of the model if not carefully selected. This study tested two sources of priors of r, and significant changes in the posterior r and the reference points determined by different runs for both cases could be observed. The abundance indices presented in this study also influenced the final results of the CMSY++ model, as results from the CMSY (catch-only) model generally differed from those presented by the BSMs. When CMSY++ ran with catch-only data it indicated a healthy stock status for the South Atlantic blue shark. However, when abundance indices were added to the catch data, most runs indicated a decline in biomass and an increase in overfishing levels for the stock. Though in our investigation we saw that the outputs given by the CMSY (catch-only) method indicated a healthy stock level, these outputs also showed exponential increases in fishing efforts in recent years, which may lead to overfishing and consequently an overfished state if the present level of fishing continues. Three BSM runs have already indicated that the South Atlantic blue shark is overfished and witnessing overfishing.

The fishery status of the South Atlantic blue shark was evaluated using different inputs of informative data applied in the CMSY++ approach. We limited the use of default settings as inputs in different runs since this is not advisable when using catch-only approaches [16]. Thus, prior estimates on r and biomass depletion rates were defined based on available knowledge of the stock. In this study, we tested runs using priors of r obtained from FishBase [21] and the prior used in the 2015 blue shark assessment report [7]. We found that priors of r in both cases for all runs indicated a healthy stock status with increasing fishing pressure when using the CMSY (catch-only) method, and almost all runs by the BSM indicated the overfishing and overfished status of the stock. We also observed a significant difference in the estimated posterior of r for both cases, especially for runs having catch and abundance indices; this change eventually influenced the reference points used in defining stock status (Table 4). When r = 0.21 [7] was used, our posterior r values for all runs tested (0.118–0.237) fell within the estimated range of values obtained in the ICCAT blue shark assessment report [7]. This range of resilience indicating a low to medium level of stock exploitation [15] shows increasing fishing pressure on South Atlantic blue sharks. Therefore, we note the importance of reliable priors when using CMSY++ to obtain good estimates of depletion in the final year, since this model gave results close to past assessment results when we applied a prior of r that closely depicted the South Atlantic blue shark stock.

This study also showed that outputs from CMSY++ can be influenced when different CPUE indices are combined with catch data. Among all abundance indices used, only the BSM run of the Brazil CPUE index had a similar output outcome to the catch-only method run, with the BSMs of the other indices indicating varying results from the CMSY runs. For example, the CMSY (catch-only) and the Brazilian BSM runs both indicated that the blue shark stock was in a healthy state, while the other runs either stated that the stock was currently either witnessing overfishing or was overfished and witnessing overfishing at the same time. The Japanese, Uruguayan and Spanish BSM runs indicated that overfishing may be occurring and that the stock may also be overfished.

Compared to the assessment methods used in the 2015 South Atlantic blue shark assessment meeting, our results correlate more closely with the runs from the state-space production model in JAGS than with the Bayesian Surplus Production (BSP) model. Though model runs in the state-space production model used in the 2015 assessment meeting had combined CPUE indices with three different process errors (0.05, 0.01 and 0), most runs indicated that the stock could be overfished (B2013/Bmsy = 0.78 to 1.29 against B2020/Bmsy = 0.623 to 1.15) and that overfishing could be occurring (F2013/Fmsy = 0.54 to 1.19 against F2020/Fmsy = 0.818 to 1.78). The scenarios with the BSP model indicated that the stock was not overfished (B2013/Bmsy = 1.96 to 2.03) and that overfishing was not occurring (F2013/Fmsy = 0.01 to 0.11). The BSP results greatly differed from the present study, which may be due to the configuration and assumptions of the models used and also maybe the shorter catch time series used in the BSP. The conclusions from the 2015 assessment meeting were that the estimates obtained with the state-space BSP were generally less optimistic, given that their outputs changed and had more pessimistic results, especially when process errors were not included [7]. Process variations may indicate changes in biomass that differ from Schaefer model expectations; this may be due to strong environmental variation, CPUEs not properly describing the abundance, or the priors used being mis-specified [15]. Our study showed minimal changes in process deviations for all model runs, indicating that our results may have better outcomes.

As indicated in [15,16], catch-only methods, including CMSY++, could have broad application in achieving fisheries’ sustainable development goals at national as well as regional fishery management levels, given their performances and their flexible usage. However, discrepancies in results may arise when using catch-only models without good knowledge of priors, as was observed in the present study when some prior information was obtained from FishBase (in our study, prior of r). Some past studies also support our results, stating that discrepancies may arise when using catch-only methods for evaluations when using life–history meta-analyses from platforms such as FishBase [16,26,27]. As seen in the present study, the default setting of r (from FishBase, Table 3) resulted in posterior resilience, indicating a very low depletion biomass state of the stock; this was a distorted picture of the evolution of the stock, given that the blue shark population in the South Atlantic is currently declining, thus corresponding to medium to high biomass exploitation levels [4,15].

## 5. Conclusions

When using informative priors of initial depletion, especially the prior of r combined with a well-defined index of abundance, CMSY++ model performance improved, depicting clearer biomass trends of blue shark stock. Besides catch time series start years, abundance indices and biological prior information, the potential performance of stock assessment models may also be affected by factors such as gear efficiency [26]. Although the usage of CMSY++ in the present study showed various limitations when comparing outputs to the BSP model used in 2015, we still think some positive and probably fairly robust information can be obtained from the analysis that could be helpful to further guide the implementation of CMSY++ to other stocks, and also to present relevant information on the stock status of blue sharks in the South Atlantic Ocean.

## Figures and Tables

**Table 1:** Ranges of different categories of the intrinsic growth rate or resilience (r) and the depletion rate or biomass relative to the unfished stock (B/k).

**Resilience/Intrinsic Growth Rate (r)**	**Prior r Range**
High	0.6–1.5
Medium	0.2–0.8
Low	0.05–0.5
Very low	0.015–0.1
**Depletion rate**	**Prior relative biomass (B/k) range**
Very strong depletion	0.01–0.2
Strong depletion	0.01–0.4
Medium depletion	0.2–0.6
Low depletion	0.4–0.8
Nearly unexploited	0.75–1.0

**Table 2:** Prior values tested using CMSY++ (14 runs). Each run is represented by different catch per unit effort (CPUE) types (BSH_ATS: blue shark Atlantic South with reconstructed catch dataset from 1971; BSH_ATS_n: blue shark Atlantic South with nominal reported catch dataset from 1992; UR: Uruguay; BR: Brazil; JP: Japan; ESP: Spain; CHTP: Taiwan, China; and JCPUE: combined CPUE.

Run	CPUE	Start Year	End Year	r.Low	r.Hi	stb. Low	stb. Hi	intb. Yr	intb. Low	intb. Hi	endb. Low	endb. Hi	Btype	force. Cmsy	Process Error
1	BSH_ATS	1971	2020	0.045	0.3	0.4	0.8	1995	0.2	0.9	0.1	0.7	None	T	0.05
2	BSH_ATS_CPUE_UR	1971	2020	0.045	0.3	0.4	0.8	1995	0.2	0.9	0.1	0.7	CPUE	F	0.05
3	BSH_ATS_CPUE_BR	1971	2020	0.045	0.3	0.4	0.8	1995	0.2	0.9	0.1	0.7	CPUE	F	0.05
4	BSH_ATS_CPUE_JP	1971	2020	0.045	0.3	0.4	0.8	1995	0.2	0.9	0.1	0.7	CPUE	F	0.05
5	BSH_ATS_CPUE_ESP	1971	2020	0.045	0.3	0.4	0.8	1995	0.2	0.9	0.1	0.7	CPUE	F	0.05
6	BSH_ATS_CPUE_CHTP	1971	2020	0.045	0.3	0.4	0.8	1995	0.2	0.9	0.1	0.7	CPUE	F	0.05
7	BSH_ATS_JCPUE	1971	2020	0.045	0.3	0.4	0.8	1995	0.2	0.9	0.1	0.7	CPUE	F	0.05
8	BSH_ATS_n	1992	2020	0.045	0.3	0.4	0.8	2011	0.2	0.9	0.1	0.7	None	T	0.05
9	BSH_ATS_n_CPUE_UR	1992	2020	0.045	0.3	0.4	0.8	2011	0.2	0.9	0.1	0.7	CPUE	F	0.05
10	BSH_ATS_n_CPUE_BR	1992	2020	0.045	0.3	0.4	0.8	2011	0.2	0.9	0.1	0.7	CPUE	F	0.05
11	BSH_ATS_n_CPUE_JP	1992	2020	0.045	0.3	0.4	0.8	2011	0.2	0.9	0.1	0.7	CPUE	F	0.05
12	BSH_ATS_n_CPUE_ESP	1992	2020	0.045	0.1	0.4	0.8	2011	0.2	0.9	0.1	0.7	CPUE	F	0.05
13	BSH_ATS_n_CPUE_CHTP	1992	2020	0.045	0.1	0.4	0.8	2011	0.2	0.9	0.1	0.7	CPUE	F	0.05
14	BSH_ATS_n_JCPUE	1992	2020	0.045	0.1	0.4	0.8	2011	0.2	0.9	0.1	0.7	CPUE	F	0.05

**Note**: r.Low/Hi: the prior range of intrinsic growth rate for the species; stb.Low/Hi: the prior biomass range relative to the unexploited biomass (B/k) at the beginning of the catch time series; intb.Yr: a year in the time series for an intermediate biomass level; intb.Low/Hi: the estimated intermediate relative biomass range; endb.Low/Hi: the prior relative biomass (B/k) range at the end of the catch time series; btype: the type of information in the bt column of the catch file.

**Table 3:** A summary of the results from CMSY++ (catch-only and BSM) runs including catch-per-unit effort (CPUE) indices from Uruguay (UR), Brazil (BR), Japan (JP), Spain (ESP), Taiwan, China (CHTP), and combined CPUE (J_CPUE). Intrinsic growth rate r of 0.06 (0.045–0.10) obtained from FishBase [21]. Runs 1 to 7 CPUE indices denoted n_CPUE for Uruguay (UR), Brazil (BR), Japan (JP), Spain (ESP), Taiwan, China (CHTP), and combined CPUE (J_CPUE) represent runs used with nominal reported catches from 1992. r represents resilience or intrinsic growth rate; K—maximum stock size or carrying capacity; MSY— maximum sustainable yield; B—biomass level; F—fishing mortality rate; Bmsy—biomass at MSY level; Fmsy—fishing mortality at MSY level; B/Bmsy—biomass relative to Bmsy; F/Fmsy—fishing mortality relative to Fmsy.

Run	CPUE	Start Year	End Year	r	K	MSY	B	Bmsy	B/Bmsy	F	Fmsy	F/Fmsy
1	BSH_ATS_n	1992	2020	0.062	2452	37.9	1645	1226	1.2	0.021	0.031	0.716
2	BSH_ATS_n_CPUE_UR	1992	2020	0.073	1312	23.9	660	656	1	0.051	0.036	1.42
3	BSH_ATS_n_CPUE_BR	1992	2020	0.072	1484	27	869	742	1.18	0.039	0.036	1.08
4	BSH_ATS_n_CPUE_JP	1992	2020	0.064	1932	30.7	924	966	0.976	0.037	0.032	1.15
5	BSH_ATS_n_CPUE_ESP	1992	2020	0.074	1281	23.7	659	641	1.04	0.051	0.036	1.39
6	BSH_ATS_n_CPUE_CHTP	1992	2020	0.068	1673	28.7	959	836	1.15	0.035	0.034	1.04
7	BSH_ATS_n_JCPUE	1992	2020	0.072	1528	27.7	910	764	1.2	0.037	0.036	1.03
8	BSH_ATS	1971	2020	0.061	2516	38.4	1592	1258	1.16	0.021	0.031	0.753
9	BSH_ATS_CPUE_UR	1971	2020	0.074	1276	24	597	638	0.942	0.057	0.037	1.5
10	BSH_ATS_CPUE_BR	1971	2020	0.071	1607	28.5	911	803	1.14	0.037	0.035	1.05
11	BSH_ATS_CPUE_JP	1971	2020	0.069	1728	29.7	512	864	0.594	0.067	0.035	1.94
12	BSH_ATS_CPUE_ESP	1971	2020	0.074	1235	23.1	559	618	0.913	0.061	0.037	1.62
13	BSH_ATS_CPUE_CHTP	1971	2020	0.067	1675	28.3	872	838	1.04	0.039	0.033	1.16
14	BSH_ATS_JCPUE	1971	2020	0.075	1319	24.9	716	660	1.1	0.047	0.037	1.26

**Table 4:** A summary of the results from CMSY++ (catch-only and BSM) runs including catch-per-unit effort (CPUE) indices from Uruguay (UR), Brazil (BR), Japan (JP), Spain (ESP), Taiwan, China (CHTP), and combined CPUE (J_CPUE). Intrinsic growth rate bounds of (0.045–0.3) from r = 0.21 used in the 2015 blue shark assessment [20]. Runs 1 to 7 CPUE indices denote n_CPUE for Uruguay (UR), Brazil (BR), Japan (JP), Spain (ESP), Taiwan, China (CHTP), and combined CPUE (J_CPUE) with runs used with nominal reported catches from 1992. r represents resilience or intrinsic growth rate; K—maximum stock size or carrying capacity; MSY—maximum sustainable Yield; B—biomass level; F—fishing mortality rate; Bmsy—biomass at MSY level; Fmsy—fishing mortality at MSY level; B/Bmsy—biomass relative to Bmsy; F/Fmsy—fishing mortality relative to Fmsy.

Run	CPUE	Start Year	End Year	r	K	MSY	Bmsy	B	B/Bmsy	F	Fmsy	F/Fmsy
1	BSH_ATS_n	1992	2020	0.074	2027	37.5	1014	1325	1.16	0.026	0.037	0.823
2	BSH_ATS_n_CPUE_UR	1992	2020	0.257	368	24.4	184	178	0.97	0.191	0.128	1.45
3	BSH_ATS_n_CPUE_BR	1992	2020	0.142	815	29	408	494	1.22	0.068	0.071	0.972
4	BSH_ATS_n_CPUE_JP	1992	2020	0.075	1663	31.3	832	795	0.951	0.043	0.038	1.15
5	BSH_ATS_n_CPUE_ESP	1992	2020	0.223	415	23.8	207	202	0.974	0.171	0.111	1.47
6	BSH_ATS_n_CPUE_CHTP	1992	2020	0.11	1046	28.7	523	605	1.17	0.056	0.055	1.02
7	BSH_ATS_n_JCPUE	1992	2020	0.159	723	28.7	361	438	1.21	0.077	0.079	1.01
8	BSH_ATS	1971	2020	0.072	2065	37.3	1032	1345	1.15	0.025	0.036	0.818
9	BSH_ATS_CPUE_UR	1971	2020	0.227	441	25.3	220	193	0.903	0.177	0.114	1.51
10	BSH_ATS_CPUE_BR	1971	2020	0.118	1036	30.5	518	613	1.2	0.055	0.06	0.944
11	BSH_ATS_CPUE_JP	1971	2020	0.119	1049	30.8	525	322	0.623	0.105	0.057	1.78
12	BSH_ATS_CPUE_ESP	1971	2020	0.237	413	24.6	206	160	0.8	0.212	0.118	1.75
13	BSH_ATS_CPUE_CHTP	1971	2020	0.126	907	28.4	453	509	1.12	0.067	0.063	1.07
14	BSH_ATS_JCPUE	1971	2020	0.154	724	27.9	362	409	1.15	0.084	0.077	1.07

## Data Availability

Data used in this study is publicly available from the ICCAT database.
